# Supplementary material for: Changes in Disposition of Ezetimibe and Its Active Metabolites Induced by Impaired Hepatic Function: The Influence of Enzyme and Transporter Activities
Source: Pharmaceutics. 2022 Dec 8;14(12):2743. doi: 10.3390/pharmaceutics14122743 (PMC9785202; doi:10.3390/pharmaceutics14122743)
Supplement: Supplementary file 1 [file pharmaceutics-14-02743-s001.zip › pharmaceutics-2049808-supplementary.pdf]

Supplemental Materials

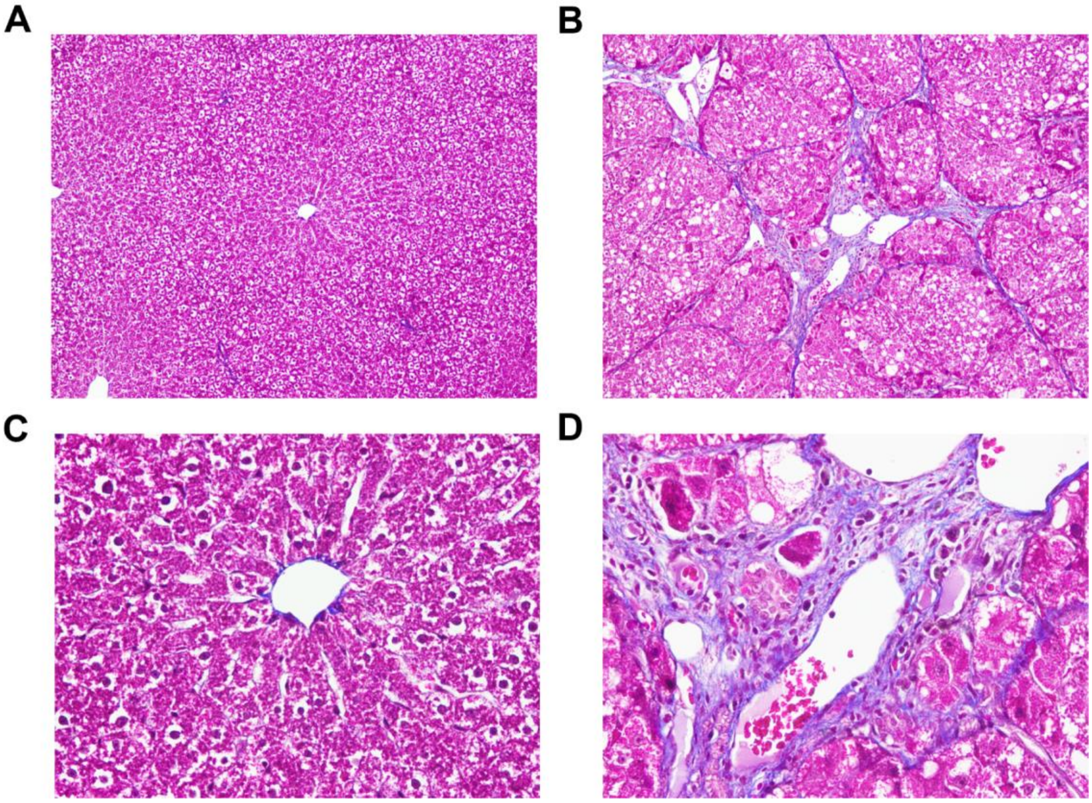

Figure S1. Histology of liver tissue from control (A: 100 ×, C: 400 ×) and CCl_4-induced (B: 100 ×, D: 400 ×) rats using masson's staining.

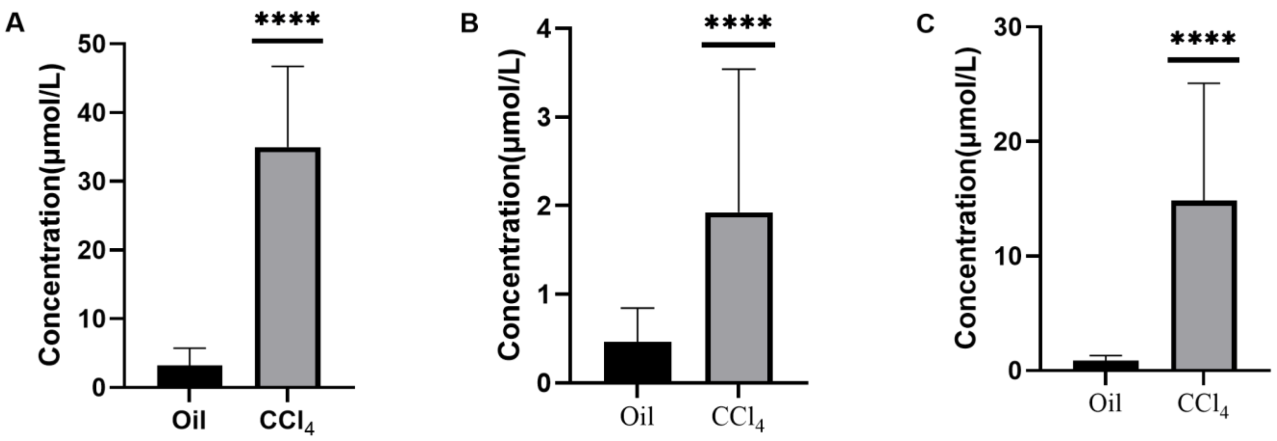

Figure S2. Concentrations of CA (A), GCA (B), and TCA (C) in the serum of control (*n* = 12) and CCl_4-induced (*n* = 12) rats. Data are expressed as mean ± SD. ****, $p < 0.0001$ compared with control.

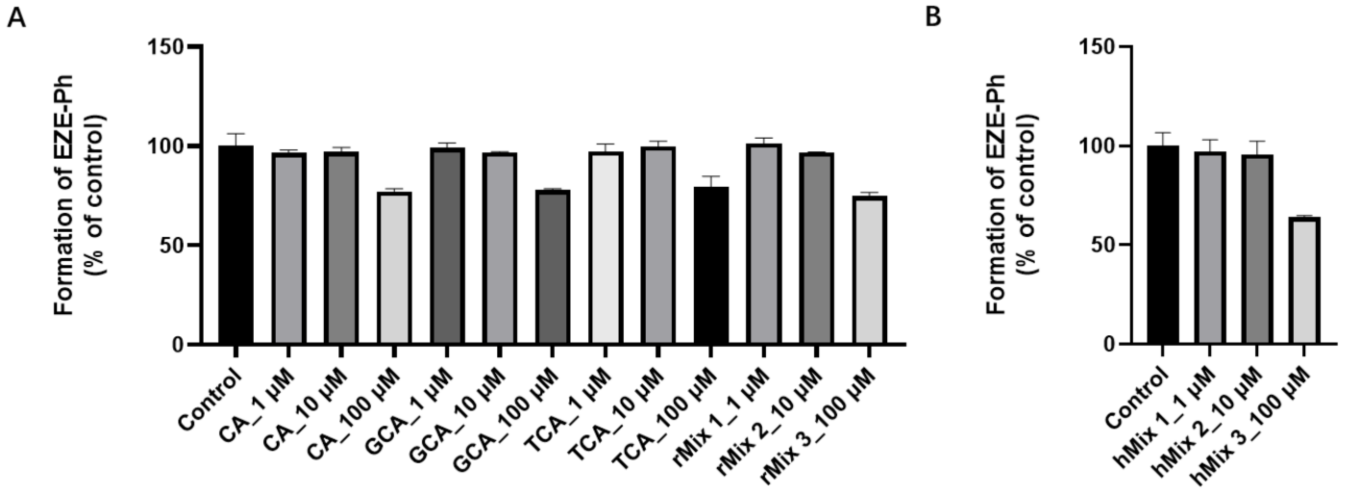

Figure S3. (A) Inhibitory effects of CA, GCA, TCA, and their mixtures on the metabolism of EZE by liver S9 from normal rats, and (B) inhibitory effects of bile acid mixtures on the metabolism of EZE by human liver S9. Data are expressed as mean ± S.D. (n = 3).

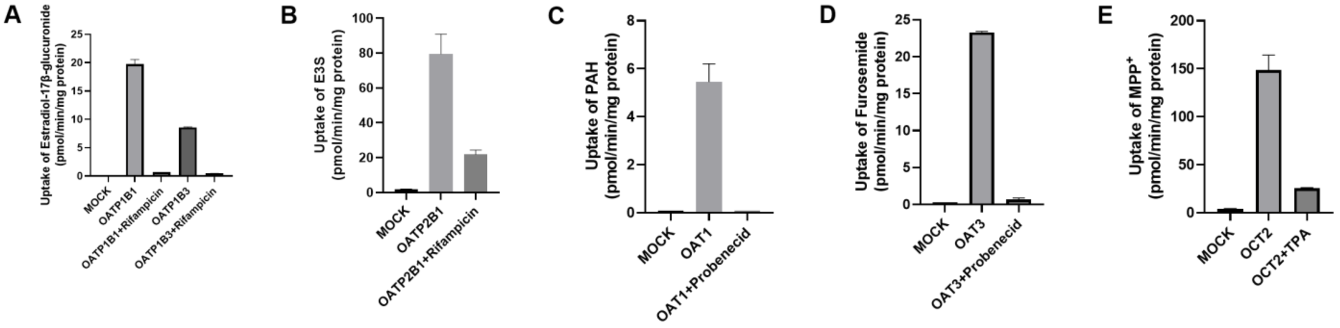

Figure S4. Uptake of estradiol-17β-glucuronide (A), E3S (B), PAH (C), furosemide (D) and MPP^+ (E) in HEK293-OATP1B1, HEK293-OATP1B3, HEK293-OATP2B1, HEK293-OAT1, HEK293-OAT3 and HEK293-OCT2, correspondingly. Data are expressed as mean ± S.D. (n = 3)

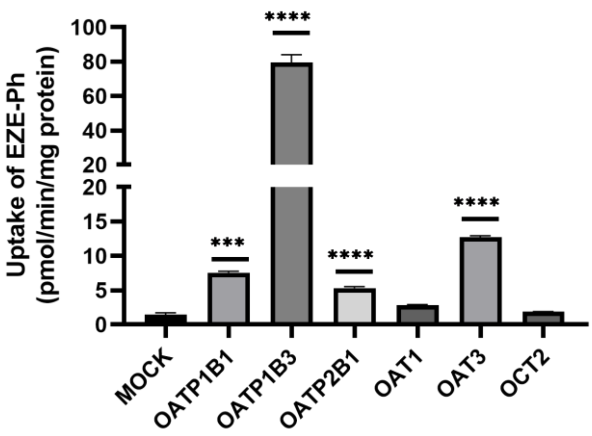

Figure S5. Uptake of 10 μM EZE-Ph in OATP1B1, OATP1B3, OATP2B1, OAT1, OAT3, and OCT2 overexpressed HEK293 cells. Data are expressed as mean ± S.D. (*n* = 3). ***$p < 0.001$, ****$p < 0.0001$ compared with control.

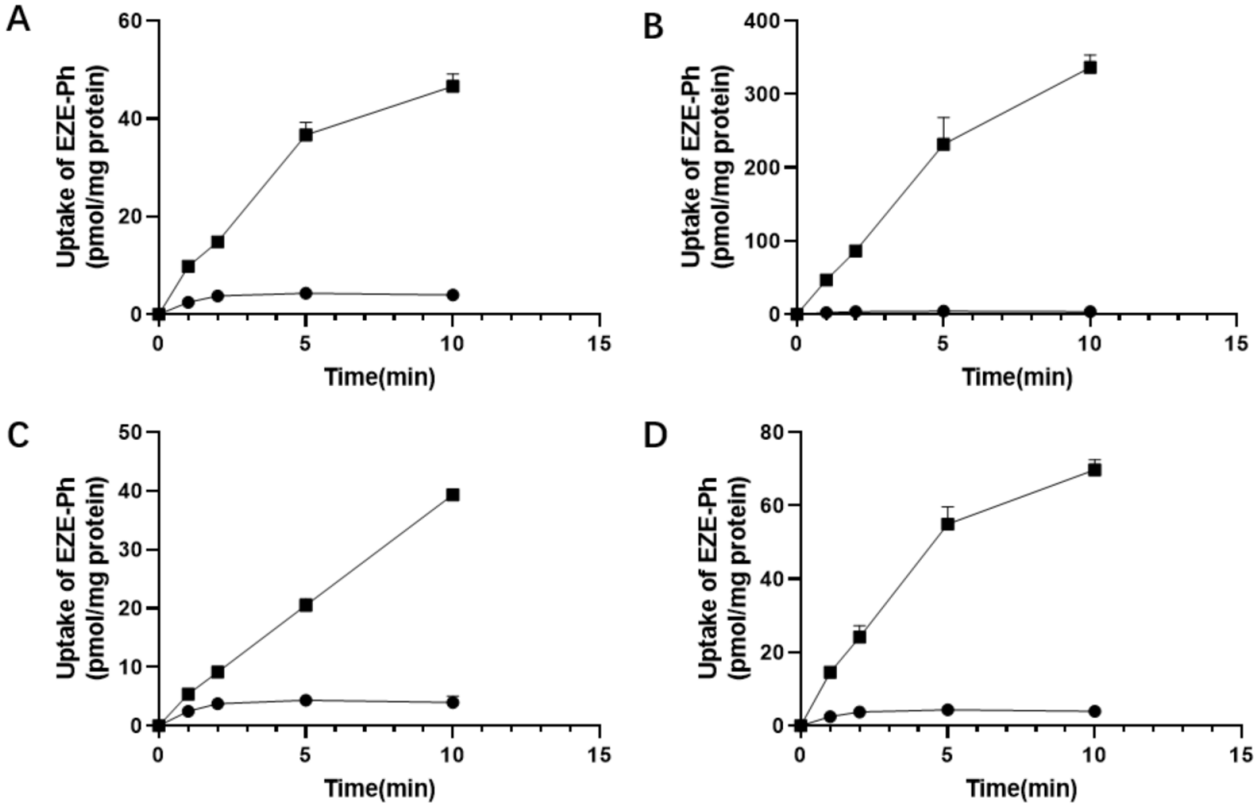

Figure S6. Time course of EZE-Ph uptake by OATP1B1-, OATP1B3-, OATP2B1- and OAT3-transfected HEK293 cells (■) and mock-transfected HEK293 cells (●). Data are expressed as mean ± S.D (n = 3). (A) Uptake of EZE-Ph by OATP1B1; (B) Uptake of EZE-Ph by OATP1B3; (C) Uptake of EZE-Ph by OATP2B1; (D) Uptake of EZE-Ph by OAT3.

Intra- and inter-run precision and accuracy data of ezetimibe and its metabolites are summarized in Table S1. The RSD values of intra- and inter-run precision were ≤8.0% and ≤11.3%, respectively; while the RE values of intra- and inter-run accuracy were within -3.5 ~ 7.2% and -3.1 ~ 7.4%, respectively.

Table S1. Intra- and inter-run precision and accuracy analysis of ezetimibe and its metabolites. Data are expressed as mean; Intra-run (n = 6, in a single run); Inter-run (n = 6, on three different runs).

**Analyte**	**Nominal Concentration (ng/mL)**	**Intra-Day**		**Inter-Day**	
		**RSD (%)**	**RE (%)**	**RSD (%)**	**RE (%)**
EZE	1.00	8.0	2.7	8.6	0.9
	3.00	5.2	-3.5	6.0	-2.6
	30.0	1.2	-3.4	2.2	-3.1
	400	1.9	-3.1	2.9	-0.4
	800	3.3	0.6	3.2	1.8
EZE-Ph	3.00	6.7	3.7	7.6	4.6
	9.00	4.4	2.6	3.5	4.7
	90.0	0.5	0.0	3.7	3.4
	1200	2.2	3.1	2.8	4.4
	2400	2.8	0.6	4.4	1.0
EZE-Hy	1.00	4.8	-0.1	11.3	-0.6
	3.00	4.3	7.2	6.8	7.4
	30.0	2.6	1.5	4.8	2.9
	400	3.2	1.8	4.0	2.5
	800	3.0	3.2	4.7	3.9

Matrix effects data of ezetimibe and its metabolites are summarized in Table S2. The IS normalized matrix factors ranged from 95.4% to 105.7% for all the analytes and the RSD values of the IS normalized matrix factors from six different sources was ≤7.7%. The RE values of the hemolytic matrix effect ranged from -11.2% to -2.5% for all the analytes and the RSD values of the hemolytic matrix effect was ≤8.4%.

Table S2. The matrix effect of ezetimibe and its metabolites.

Analyte	Nominal Concentration (ng/mL)	Internal Standard-Normalized Matrix Factor		Hemolytic Matrix Effect		
		Mean (%)	RSD (%)	Measured (ng/mL)	RSD (%)	RE (%)
EZE	3.00	98.9	4.7	2.67 ± 0.10	3.7	-11.2
	800	100.2	1.8	780 ± 22.9	2.9	-2.5
EZE-Ph	9.00	105.7	3.5	8.52 ± 0.13	1.6	-5.3
	2400	104.4	2.2	2305 ± 56.8	2.5	-4.0
EZE-Hy	3.00	95.4	7.7	2.83 ± 0.24	8.4	-5.8
	800	100.1	2.0	763 ± 20.6	2.7	-4.6

Stability data of ezetimibe and its metabolites are summarized in Table S3. EZE, EZE-Ph and EZE-Hy were stable in rat plasma under the test conditions for the LQC and HQC samples, including short-term storage at room temperature, auto-sampler and room temperature storage after sample extraction, and three freeze/thaw cycles. They also were stable in blood when left at room temperature for 2 h.

Table S3. Summary of the stability of ezetimibe and its metabolites in rat plasma (*n* = 6).

**Condition**		**EZE**		**EZE-Ph**		**EZE-Hy**	
		**LQC**	**HQC**	**LQC**	**HQC**	**LQC**	**HQC**
Short-term (room temperature 3 h)	RE (%)	-11.2	-5.6	-1.9	0.3	-6.9	-3.6
	RSD (%)	2.5	2.6	8.6	2.6	11.4	2.6
Post-extraction (room temperature 3 h)	RE (%)	0.7	3.9	8.8	5.1	8.9	5.2
	RSD (%)	3.0	2.4	2.0	3.2	10.3	2.6
Autosampler (4°C, 24 h)	RE (%)	0.6	3.9	5.3	6.0	7.9	9.0
	RSD (%)	3.8	2.1	3.5	1.7	6.9	3.2
Freeze-thaw (−20°C, three cycles)	RE (%)	-1.0	1.1	0.8	-0.4	-0.1	2.0
	RSD (%)	5.8	4.6	3.6	3.1	6.7	3.3

The concentrations of EZE/EZE-Ph/EZE-Hy at LQC and HQC were 3.00/9.00/3.00 ng/ml and 800/2400/800 ng/ml, respectively. HQC: High quality control; LQC: Low quality control; RE: Relative error; RSD: Relative standard deviation.

For dilution integrity, the RE of the 5-fold dilution samples was -3.6% for EZE, 1.8% for EZE-Ph, and -4.8% for EZE-Hy. The RSD was ≤6.2% for all the analytes, suggesting that samples exceeding the ULOQ concentration could be quantified using a 5-fold dilution.

Table S4. The components of rMix 1~3.

	**rMix 1**	**rMix 2**	**rMix 3**
CA	0.714	7.14	71.4
GCA	0.143	1.43	14.3
TCA	0.143	1.43	14.3
TBA	1.00	10.0	100

Table S5. The components of hMix 1~3.

	**hMix 1**	**hMix 2**	**hMix 3**
CA	0.0179	0.179	1.79
GCA	0.179	1.79	17.9
TCA	0.0893	0.893	8.93
CDCA	0.0357	0.357	3.57
GCDCA	0.357	3.57	35.7
TCDCA	0.179	1.79	17.9
UDCA	0.0204	0.204	2.04
GUDCA	0.102	1.02	10.2
TUDCA	0.0204	0.204	2.04
TBA	1.00	10.0	100
